# Distinct mechanisms drive divergent phenotypes in hypertrophic and dilated cardiomyopathy–associated *TPM1* variants

**DOI:** 10.1172/JCI179135

**Published:** 2024-12-16

**Authors:** Saiti S. Halder, Michael J. Rynkiewicz, Lynne Kim, Meaghan E. Barry, Ahmed G.A. Zied, Lorenzo R. Sewanan, Jonathan A. Kirk, Jeffrey R. Moore, William J. Lehman, Stuart G. Campbell

**Affiliations:** 1 Department of Biomedical Engineering, Yale University, New Haven, Connecticut, USA.; 2 Department of Pharmacology, Physiology & Biophysics, Boston University Chobanian & Avedisian School of Medicine, Boston, Massachusetts, USA.; 3 Department of Biological Sciences, University of Massachusetts Lowell, Lowell, Massachusetts, USA.; 4 Department of Cell and Molecular Physiology, Stritch School of Medicine, Loyola University Chicago, Chicago, Illinois, USA.

**Keywords:** Cardiology, Cardiovascular disease

## Abstract

Heritable forms of hypertrophic cardiomyopathy (HCM) and dilated cardiomyopathy (DCM) represent starkly diverging clinical phenotypes, yet may be caused by mutations to the same sarcomeric protein. The precise mechanisms by which point mutations within the same gene bring about phenotypic diversity remain unclear. Our objective was to develop a mechanistic explanation of diverging phenotypes in two *TPM1* mutations, E62Q (HCM) and E54K (DCM). Drawing on data from the literature and experiments with stem cell–derived cardiomyocytes expressing the *TPM1* mutations of interest, we constructed computational simulations that provide plausible explanations of the distinct muscle contractility caused by each variant. In E62Q, increased calcium sensitivity and hypercontractility was explained most accurately by a reduction in effective molecular stiffness of tropomyosin and alterations in its interactions with the actin thin filament that favor the “closed” regulatory state. By contrast, the E54K mutation appeared to act via long-range allosteric interactions to increase the association rate of the C-terminal troponin I mobile domain to tropomyosin/actin. These mutation-linked molecular events produced diverging alterations in gene expression that can be observed in human engineered heart tissues. Modulators of myosin activity confirmed our proposed mechanisms by rescuing normal contractile behavior in accordance with predictions.

## Abstract 2

This manuscript enables revealing insights into the mechanisms by which spatially proximate mutations to the same gene can lead the myocardium to very different disease states using a variety of computational and experimental techniques.

## Graphical abstract

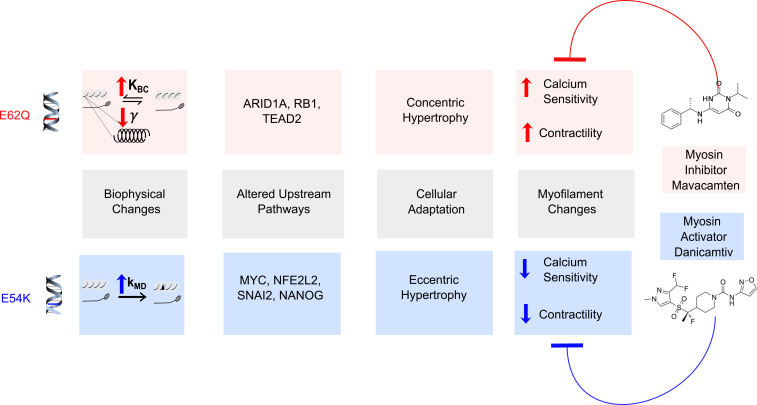

## Introduction

Hypertrophic cardiomyopathy (HCM) and dilated cardiomyopathy (DCM) are two of the most common forms of genetic heart diseases and are a leading cause of sudden death and heart failure in otherwise young and healthy individuals (1). HCM is associated with thickening of the left ventricular wall, myocyte disarray, and interstitial fibrosis (2), while DCM is a condition defined by thinning of the left ventricular wall, enlargement of the left ventricular chamber, reduced ejection fraction, and fibrosis (3). Despite being phenotypically divergent, both diseases have been linked to mutations in many of the same sarcomeric proteins of the thick and thin filaments. For instance, the R403Q, R453C, and K207N mutations in *MYH7* (myosin heavy chain) have been shown to cause HCM, while S532P and F764L mutations in the same gene have been associated with DCM phenotypes (4). Similarly, over 30 HCM-causing mutations and 10 DCM-causing mutations have been identified in *TNNT2* (5), some of which are linked to the same protein residue. In the gene encoding α-tropomyosin (*TPM1*), the disease phenotypes associated with identified mutations are divided almost evenly between HCM and DCM.

Thus, considerable effort has been spent in attempting to distinguish the acute physiological effects of HCM versus DCM mutations. In vitro motility assays using purified proteins, skinned muscle fiber preparations, or membrane-permeabilized cells have been used frequently to identify and categorize functional differences caused by HCM or DCM mutations at an in vitro scale. These studies generally suggest that HCM mutations increase calcium sensitivity of actin/myosin activity while DCM mutations decrease it (6). However, there are exceptions to this paradigm. For example, these assays have shown that both R312C (HCM-causing) and R312H (DCM-causing) mutants in cardiac actin reduce calcium sensitivity (7). In another case, high doses of the HCM-causing mutant *TNNT2* R278C administered to membrane-permeabilized cells resulted in a rightward shift in force-pCa curve — where pCa is defined as the negative logarithm of calcium ion concentration — indicating a decrease in calcium sensitivity (8). Therefore, alterations in calcium sensitivity are often not clearly predictive of disease phenotype.

In contrast, an arguably more physiologically relevant study in transgenic mice by Davis and colleagues generated a more consistent picture of HCM versus DCM pathogenesis. Using transgenic mice expressing either HCM- or DCM-causing cTnC mutations, they measured the force-time integral (FTI; an index computed by integration of muscle isometric twitch force-time values), which served as a reliable predictor of phenotype (9). In their examination of isolated heart muscle preparations, mutations that caused increases in FTI, without exception, caused left ventricular hypertrophy. Conversely, mutations that decreased FTI produced DCM.

To determine whether the relationship between FTI and HCM/DCM phenotypes extends to mutants in TPM1, we undertook a comparison of HCM and DCM mutations in *TPM1* to serve as an extensively characterized simple model system. The two *TPM1* mutations chosen were E54K (DCM) and E62Q (HCM) based on their pathogenicity and extensive mechanistic characterization (6, 10–13). Both are located on the same actin-binding repeat of tropomyosin (14) and positioned only 8 amino acids apart. Their formal classifications as DCM- and HCM-linked, respectively, are based on strong clinical evidence. E54K was identified as a novel mutation in 2001 in a study involving DCM patients. The familial proband died at age 27 while waiting for a heart transplant, and their father and paternal uncle died at ages 27 and 49, both from heart failure. E62Q was first identified in 2003 in a large Dutch HCM family spanning 5 generations. Nine individuals in the same family died from sudden cardiac death at varying ages ranging from 15 to 47 years.

Hence, we engineered *TPM1^E62Q/E62Q^* and *TPM1^E54K/E54K^* mutations into the same WT human induced pluripotent stem cell (iPSC) line. This enabled comparison of phenotypes on an isogenic background and in the context of human genetics. By seeding iPSC-derived cardiomyocytes into engineered heart tissues (EHTs), we were able to study physiologically relevant muscle function while excluding potentially confounding secondary effects caused by neuroendocrine control of cardiac function or other compensatory mechanisms. The choice to use *TPM1* variants for this study allowed a strong mechanistic basis on which to seek detailed explanation of mutation effects at the single-molecule and residue-to-residue levels.

Our work reveals starkly diverging phenotypes and a diverse set of differentially expressed genes in these cases of HCM versus DCM as well as specific, plausible molecular mechanisms by which the respective mutations provoke their unique phenotypes. These data and analyses confirm the notion that left ventricular hypertrophy can be driven by hypercontractility and dilation by hypocontractility, congruent with the hypothesis advanced by Davis et al. (9). Additionally, our conclusions are supported by demonstration that non-acute treatment of mutation-expressing EHTs with appropriate small molecules to counter aberrant myofilament behavior can correct disease phenotypes.

## Results

Further information can be found in Supplemental Results.

### Hyper- and hypocontractility observed in E62Q and E54K EHTs.

Three isogenic iPSC cell lines were used to study the impact of *TPM1* HCM/DCM mutations on cardiomyocyte function: WT, WT with a CRISPR/Cas9–induced homozygous *TPM1^E62Q/E62Q^* mutation (HCM-causing), and WT with a CRISPR/Cas9–induced homozygous *TPM1^E54K/E54K^* mutation (DCM-causing). These cells were then seeded onto decellularized porcine myocardium to fabricate EHTs, which we used to evaluate the active contractile properties of the EHTs.

**Figure 1:**
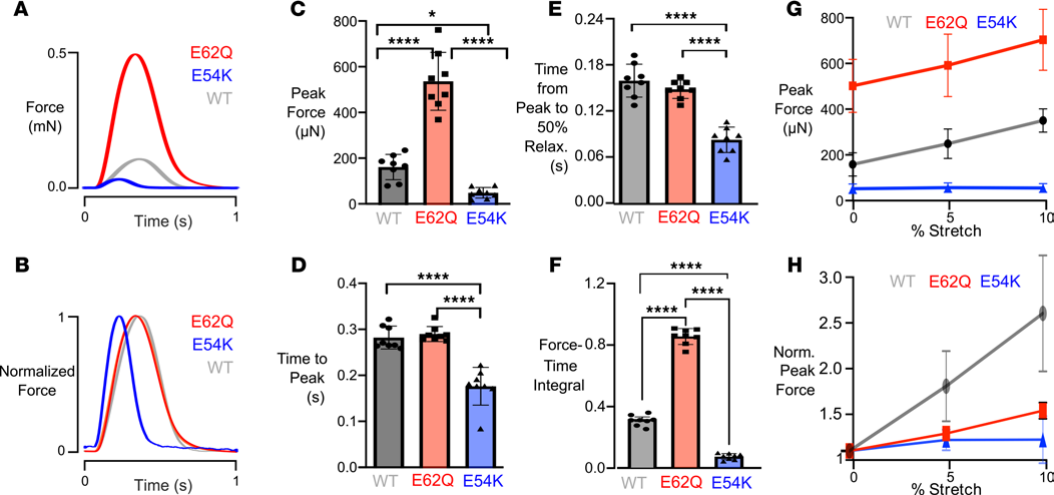
Basic twitch properties of WT, E62Q, and E54K EHTs while pacing at 1 Hz. (**A** and **B**) Sample force traces. (**C**) Peak force. (**D**) Time from start of stimulus to peak force. (**E**) Time from peak to 50% relaxation. (**F**) Force-time integral. (**G**) Length-dependent activation of EHTs showing peak forces at 0%, 5%, and 10% stretch. No statistical test was performed. (**H**) Length-dependent activation of EHTs showing normalized peak forces (data from each EHT normalized to its own peak force at culture length, i.e., 0% stretch). E62Q and E54K curves are significantly different from WT by 2-way ANOVA with multiple comparisons at 5% stretch (*P* = 0.0156 and *P* = 0.0076, respectively) and at 10% stretch (*P* = 0.051 and *P* = 0.0007, respectively).*P < 0.05, *P < 0.0001.

The mutant EHTs demonstrated drastically different isometric twitch phenotypes (Figure 1, A and B). E62Q EHTs produced a greater than 3-fold increase while E54K EHTs demonstrated an almost 3-fold decrease in the active peak force compared with WT (Figure 1C). Although the twitch kinetics did not vary significantly between E62Q and WT EHTs, E54K EHTs exhibited a shorter-lived twitch and took 42% less time than WT EHTs to reach peak force (Figure 1D) and only took half as much time as WT EHTs to relax (Figure 1E). The significant twitch kinetic differences in E54K compared with both E62Q and WT were also reflected in the reduced force-time integral (FTI) (Figure 1F). Since impaired Frank-Starling mechanism is often an indication of systolic abnormalities, we also measured the length-dependent activation of EHTs by assessing the peak force generated at various levels of tissue stretch (Figure 1G). We normalized the peak forces to each group’s mean contractile force at culture length (0% stretch) to better visualize the relative length-dependent activation (Figure 1H). We observed that both E54K and E62Q EHTs demonstrated impaired length-dependent activation compared with WT EHTs.

### Diastolic stiffness is drastically higher in E62Q EHTs.

**Figure 2:**
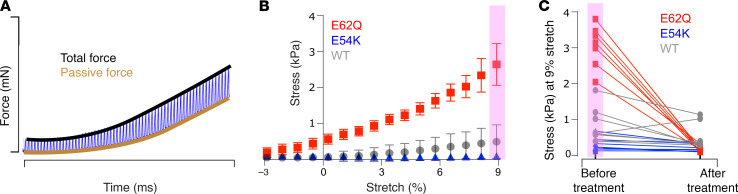
Diastolic stiffness. (**A**) Sample force trace during stretch. (**B**) Passive stress in WT, E62Q, and E54K EHTs (*n* = 8). E62Q passive stress is significantly different from E54K and WT using 2-way ANOVA. (**C**) Passive stress in WT, E62Q, and E54K EHTs at 9% stretch before and after 30 minutes of 2 μM mavacamten treatment (*n* = 8; passive stress values are significantly different using 2-way ANOVA with multiple comparisons, *P* = 0.0001; significant interaction between genotype and mavacamten treatment).

Having observed drastically different active isometric twitch properties, we chose to examine whether the biomechanical properties of tissues in between stimulated contractions (passive, or “diastolic,” properties) were also changed in the E62Q and E54K mutants. EHTs were slowly stretched from a slack length (–3% relative to culture length) to a stretch of 9%, while being electrically paced at 1 Hz (Figure 2A). The diastolic force in this case is defined as the minimum force level observed between stimulus events. E62Q EHTs showed a greater than 4-fold increase in diastolic stress across all stretch levels compared with WT, while E54K EHTs seemed to be slightly more compliant than WT EHTs, although this change was statistically insignificant (Figure 2B). We hypothesized that the increased diastolic stress may have been a result of incomplete inhibition of actin-myosin interactions during the diastolic interval. To test this, we subjected the tissues to a high concentration of mavacamten, a small-molecule myosin inhibitor, for 30 minutes and then re-measured length-dependent diastolic stresses. The acute drug treatment drastically reduced the diastolic stress in E62Q EHTs, compared with WT or E54K, resulting in a significant genotype-drug interaction (*P* = 0.0001) (Figure 2C) and indicating that the increased diastolic stress in E62Q EHTs could be attributed to the presence of residual actin-myosin cross-bridges during the diastolic interval.

### Divergent transcriptomic signatures of E62Q and E54K EHTs.

**Figure 3:**
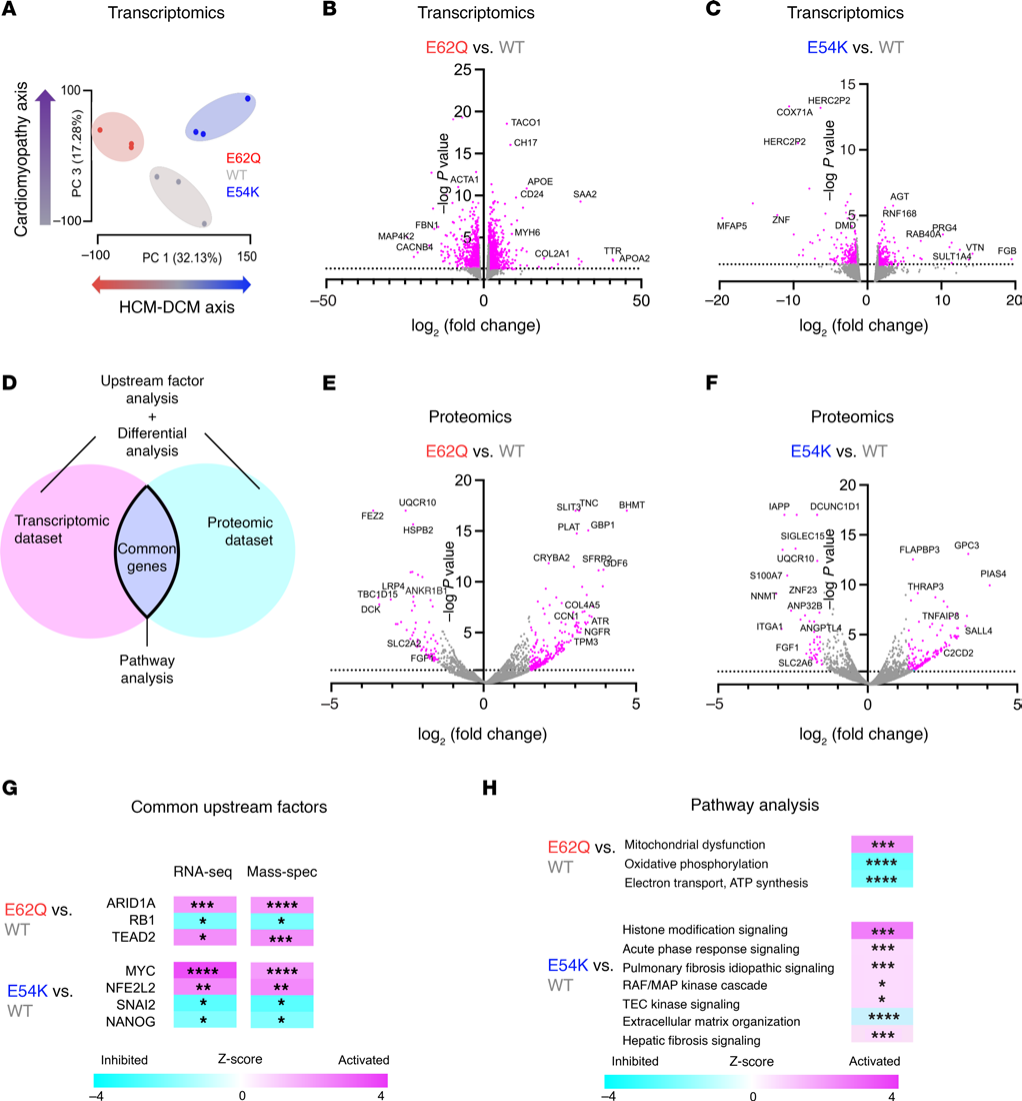
Transcriptomic and proteomic analysis of EHTs. (**A**) Exploratory principal component analysis of the transcriptomic data (bulk RNA sequencing) where each point represents a unique sample. (**B**) Volcano plots from RNA sequencing analysis of differentially regulated genes in E62Q versus WT comparison. (**C**) Volcano plots from RNA sequencing analysis of differentially regulated genes in E54K versus WT comparison. The full data set is available in the GEO database under accession ID GSE251993. (**D**) Schematic to explain how the transcriptomic and proteomic data sets were used for different types of analysis. (**E**) Volcano plots from mass spectrometry data of differentially regulated proteins in E62Q versus WT comparison. (**F**) Volcano plots from mass spectrometry data of differentially regulated proteins in E54K versus WT comparison. (**G**) Common upstream transcription factors from independent upstream analysis of proteomic and transcriptomic data. (**H**) Top canonical pathways identified by Ingenuity Pathway Analysis using common set of genes from proteomic and transcriptomic data sets. *P < 0.05, **P < 0.01, ***P <.001, ****P <.0001.

Given the very divergent phenotypes exhibited by the two *TPM1* mutants in both systolic and diastolic assessments, we next sought to identify any accompanying changes in gene expression and cell signaling pathways. To that end, we performed bulk RNA sequencing on WT, E62Q, and E54K EHTs. A principal component analysis on the resulting data showed distinct clusters formed by WT, E62Q, and E54K EHTs. We plotted the first (PC1) and third (PC3) principal components and noted that the mutant groups diverged from the WT cluster along PC1, but both shifted away from the WT cluster along PC3 (Figure 3A). This implies that PC1 represents the set of genes that are differentially expressed in HCM and DCM while PC3 represents the set of genes that are expressed differentially because of homeostatic imbalance regardless of whether the mutation is HCM- or DCM-linked. Differential expression analysis of transcriptomic data revealed that over 1,500 genes were differentially regulated in both HCM and DCM compared with WT (Figure 3, B and C). To see what proteomic changes were occurring as a result of the *TPM1* mutations, we performed mass spectrometry. Mass spectrometry also yielded over 800 differentially regulated proteins in both mutants (Figure 3, E and F). From the mass spectrometry data we queried the levels of various sarcomeric protein isoforms (Supplemental Figure 3; supplemental material available online with this article; https://doi.org/10.1172/JCI179135DS1). Our EHTs exhibited no significant changes between mutant EHTs and WT EHTs in terms of *MYH6*, *MYH7*, *TNNI1*, *TNNI3*, *TTN*, and ratio of cardiac troponin I to total troponin I and β-myosin heavy chain to total myosin. HCM EHTs had significantly higher *MYL2* compared with WT EHTs. We next sought to investigate what pathways and signaling molecules were activated in each disease. We first performed upstream factor analysis independently on the proteomic and transcriptomic data sets. We used QIAGEN Ingenuity Pathway Analysis to analyze which upstream transcription factors were activated or inhibited for each disease. Transcription factors that showed up in both independent upstream analyses are shown in Figure 3G. We then looked at the set of genes and proteins that were congruently upregulated or downregulated in the 2 data sets and then subjected this set to pathway analysis (Figure 3D). This revealed modulation of several canonical signaling pathways (Figure 3H), with HCM mutation impacting metabolic pathways and DCM mutation impacting extracellular matrix reorganization and fibrotic pathways.

### Differences in 2D cardiomyocyte morphology.

**Figure 4:**
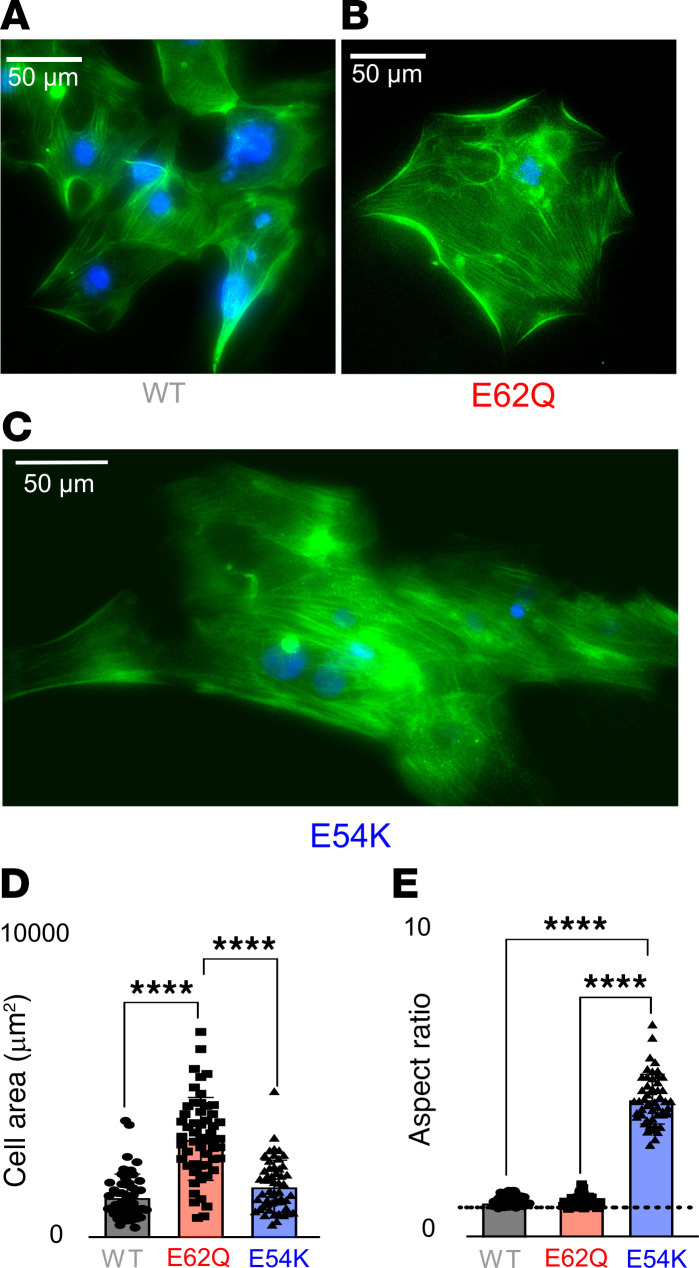
Cell size measurements. (**A**–**C**) cTnT (green) and DAPI (blue) staining of WT (**A**), E62Q (**B**), and E54K (**C**) iPSC-CM cells. Scale bars: 50 μm. (**D** and **E**) Cell area (**D**) and aspect ratio (**E**) measurements. Statistical analysis: 1-way ANOVA with multiple comparisons; *P* values for multiple comparisons are indicated using asterisks (****P < 0.0001).

Given the divergent transcriptomic signatures of the mutant EHTs, and enrichment of canonical pathways that are consistent with changes in cell morphology such as oxidative phosphorylation and mitochondrial dysfunction (15), we next sought to investigate whether cell morphology was changing in our disease models as well. Because it is difficult to calculate cell size accurately when the cells are seeded onto scaffolds, we cultured differentiated cardiomyocytes in sparse 2D monolayers. We noticed that even in a simple culture milieu, there were drastic changes in the morphology of mutant cardiomyocytes (Figure 4, A–C). Upon analyzing the images, we found that the average cell area was more than 2-fold higher in E62Q cells compared with WT, while E54K cells were not significantly different in size (Figure 4D). E54K cells, however, were more elongated and had a 3-fold higher aspect ratio (ratio of major to minor axis of fitted ellipse) compared with both WT and E62Q EHTs (Figure 4E).

### Plausible mechanisms of pathogenicity in E62Q and E54K can be identified using computational models.

Having observed robustly different expressions of morphological phenotypes in 2D cardiomyocytes as well as functional phenotypes in 3D EHTs, we next aimed to identify the fundamental mechanism linking the diseased phenotypes to their respective genotypes. One well-established fact is that both these mutations alter calcium sensitivities as measured by in vitro motility assays or thin filament reconstituted skinned muscle fibers. Results from previous studies that were used in subsequent analysis are summarized in Table 1, which shows higher calcium sensitivity in E62Q and lower calcium sensitivity in E54K. We confirmed these shifts in calcium sensitivity by performing our own regulated in vitro motility assays on both mutants (Supplemental Figure 1).

**Figure 5:**
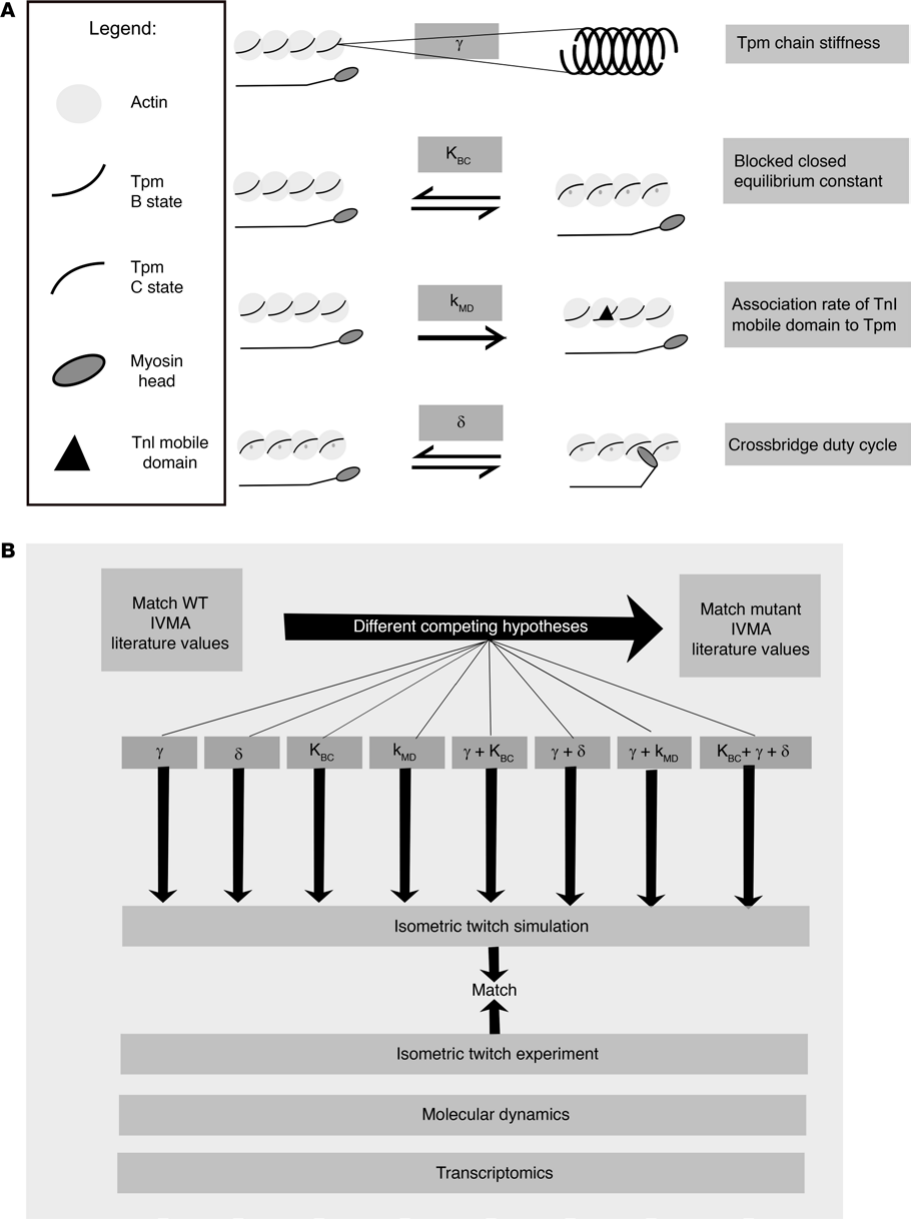
Hypothesis-driven approach. (**A**) Tunable parameters used in the simulations. (**B**) Evaluation of several competing hypotheses. TnI, troponin I; Tpm, tropomyosin; IVMA, in vitro motility assay.

To understand how the 2 mutations could plausibly exert their respective effects on myofilament calcium sensitivity, we used a 24-state Markov model of thin filament function previously published by our group (16). This model is based on current understanding of tropomyosin’s role within the thin filament regulatory switch as well as its binding interactions with other sarcomeric proteins. We encapsulate tropomyosin function in terms of 4 fundamental model parameters as follows: Tropomyosin stiffness (γ) is an intrinsic molecular property that affects cooperative spread of thin filament activation. The blocked-closed equilibrium constant (K_BC_) is determined largely by electrostatic interactions between tropomyosin and actin and describes the relative equilibrium of tropomyosin between its primary actin regulatory states. It is important to note that in this model, changes in the blocked-closed equilibrium constant (K_BC_) are implemented as adjustments in the forward kinetic rate (B to C) and, as such, appropriately modulate both steady-state and dynamic responses. The association rate of troponin I mobile domain to tropomyosin/actin (k_MD_) is a key regulatory step in Ca^2+^ activation of the thin filament. Finally, as myosin binds to the thin filament and undergoes its power stroke, it interacts with and displaces tropomyosin into its open (M) regulatory state. Hence, alterations in tropomyosin may affect the myosin cross-bridge duty cycle (δ), which is the proportion of time myosin spends attached to the thin filament during its cycle (Figure 5A). Our goal was to seek a set of mutant tropomyosin parameter changes that could simultaneously explain the steady-state and isometric twitch alterations associated with each mutant.

We first set the parameters of the model to match literature values of WT calcium sensitivity (pCa_50_) and cooperativity (n_H_). Then we applied changes in the parameters representing distinct hypotheses to see which could match literature values of calcium sensitivity (pCa_50_) and cooperativity (n_H_) associated with each mutation. Subsequently, we applied the same proportional change in parameters that provided the best description of the calcium sensitivity to simulate isometric twitches and compared the simulations with our experimental results (Figure 5B).

### Contractile phenotypic divergence in HCM- and DCM-linked mutations may be explained by different underlying mechanisms.

**Figure 6:**
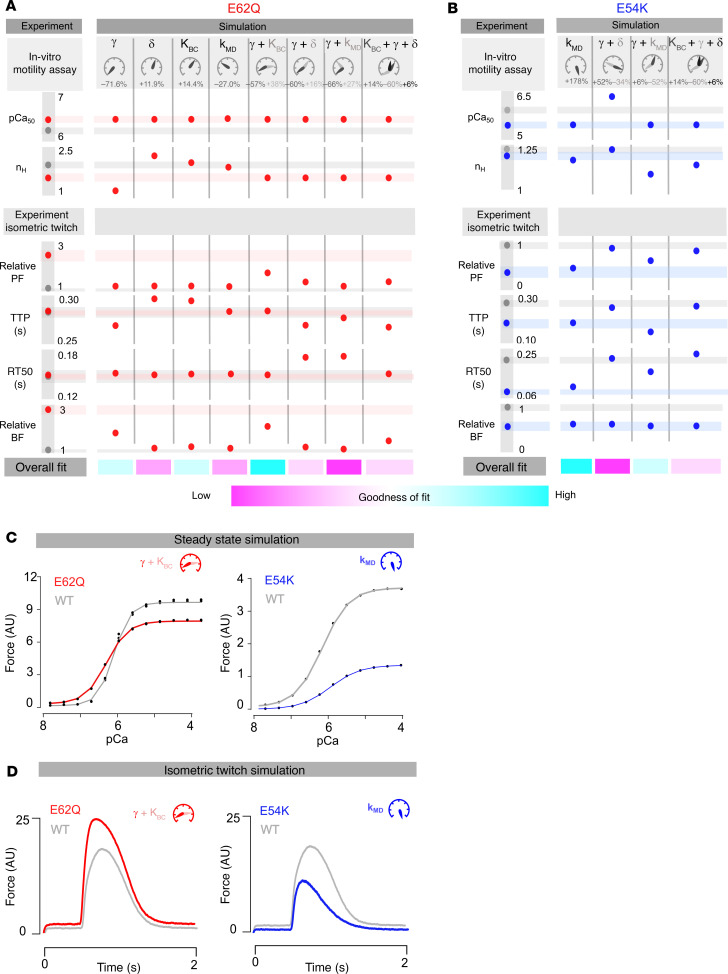
Computational simulations. Speedometer graphics show the direction and magnitude of parameter changes (not to scale). Gray, red, and blue shaded horizontal bars represent the standard deviations of experimental data sets for WT, E62Q, and E54K EHTs, respectively. Gray, red, and blue dots represent the mean of experimental data sets and output of computational simulations for WT, E62Q, and E54K EHTs, respectively. An ideal match would show dots inside all the bars of the same color. (**A**) Steady-state (top) and twitch simulation (bottom) result summary for E62Q. (**B**) Steady-state (top) and twitch simulation (bottom) result summary for E54K. (**C**) Steady-state simulations for the winning hypotheses (γ + K_BC_ for E62Q and k_MD_ for E54K) in each case. (**D**) Isometric twitch simulations for the winning hypotheses (γ + K_BC_ for E62Q and k_MD_ for E54K) in each case. PF, Peak Force; TTP, Time to Peak; RT50, Time to Relax to 50%; and BF, Baseline Force.

For each hypothesis described in Figure 5B, we changed only the parameters in question relative to the WT parameter set while leaving all other model parameters constant. The parameter values that most accurately reflected the change from WT to mutant calcium sensitivity (pCa_50_) and cooperativity (n_H_) values were recorded (Figure 6, A and B). All steady-state simulations done for E62Q yielded perfect matches with literature values for calcium sensitivity (pCa_50_) for the parameter changes shown. However, cooperativity (n_H_) values could only be matched using multi-parameter hypotheses. In the case of E54K, single-parameter changes to γ, K_BC_, or δ could not match in vitro measurements. Among the remaining hypotheses, all except the γ + δ hypothesis yielded perfect matches with literature values for calcium sensitivity (pCa_50_). In general, it was more difficult to obtain good cooperativity (n_H_) matches for E54K across all hypotheses, with only the k_MD_ providing a reasonable fit.

Having generated multiple parameter sets capable of explaining steady-state data, we sought additional refinement of candidate mechanisms by determining which might also produce reasonable predictions of isometric twitch force measurements (Figure 1). For E62Q, isometric twitch simulations of all hypotheses generated peak forces lower than measured experimental values but with the γ + K_BC_ hypothesis coming the closest with a 1.5-fold increase. Our experimental results showed no change in time to peak force (TTP) and time from peak force to 50% relaxation (RT50), a scenario that was also most accurately reflected by the γ + K_BC_ hypothesis. The γ-only, K_BC_-only, and K_BC_ + γ + δ hypotheses matched RT50 but not TTP. The 3-fold increase in baseline force measured by in vitro experiments was also most accurately reflected by the γ + K_BC_ hypothesis.

For E54K, for which experimental results showed shorter-lived and hypocontractile twitches, the k_MD_-only and k_MD_ + γ hypothesis simulations offered the closest matches across all metrics. Among these two, the k_MD_-only hypothesis recapitulated experimental results most accurately. Based on these semiquantitative comparisons, we found the best explanation for the effects of E62Q to be a decrease in tropomyosin’s molecular stiffness (γ) combined with altered tropomyosin-actin electrostatics such that the blocked-closed equilibrium (K_BC_) was shifted toward the closed state. The functional effects of E54K seem best and most simply explained by an increased affinity of tropomyosin for the cTnI mobile domain (k_MD_). The steady-state simulations and isometric twitch simulations for each winning hypothesis are shown in Figure 6, C and D, for E62Q and E54K, respectively.

### Atomistic simulations provide qualitative and quantitative evidence to support proposed mechanisms.

**Figure 7:**
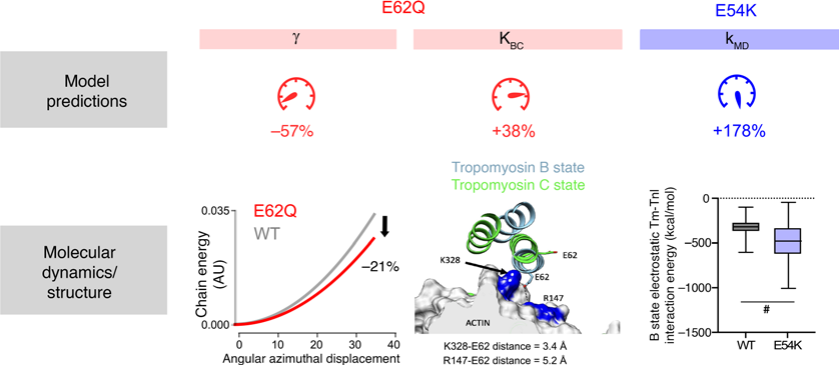
Molecular evidence supporting model predictions. Left: Chain energy versus azimuthal displacement showing decreased effective stiffness of E62Q calculated using the 2D coarse-grain model. Middle: PDB structures of E62 tropomyosin in low and high calcium. Right: Tropomyosin–troponin I (Tm-TnI) interaction energy in the B state of WT and E54K tropomyosin (averaged over *n* = 1,000 frames for WT and *n* = 3,000 frames for E54K; error bars represent standard deviation). ^#^Results significantly different using bootstrapping to analyze difference of means. In this case 11 of 10,000 repeated samplings with replacement showed no difference between means (equivalent to *P* value of 0.0011).

Having identified plausible molecular-level characteristics describing each mutation’s effect on contractile phenotypes, we sought supporting evidence by examining structural models of tropomyosin and the thin filament. Our model fits suggested a drop in tropomyosin stiffness as a potential mechanism for E62Q. We therefore used molecular dynamics simulations of the tropomyosin molecule and fed the observed local angular fluctuations along its axis into a previously published coarse-grain model (17) to estimate the effective tropomyosin chain stiffness. The chain energy calculated over a range of azimuthal displacements (Figure 7, left) demonstrated a 21% drop in tropomyosin stiffness when E62Q was introduced (compared with WT). The model predicted a 57% reduction in tropomyosin stiffness. While the magnitude of the change is different, the directionality of the change is consistent.

The other molecular change we hypothesized from the model fits to E62Q data was an increase in the blocked-closed equilibrium constant (K_BC_). We examined the Protein Data Bank structures (PDB ID: 7UTI and 7UTL) of WT tropomyosin in the presence and absence of calcium (Figure 7, middle). In the low-calcium state, tropomyosin residue E62 is close to K328 and R147 on actin. Mutating the charged residue to a polar one would presumably weaken this interaction, thus destabilizing the B state and making the filament easier to turn on (18). This is precisely what was represented in the model by an increase in the K_BC_ parameter.

For E54K, we measured the B-state electrostatic tropomyosin–troponin I interaction energies using atomistic simulations. Molecular dynamics simulations in explicit solvent were performed twice comprising 4 independent replicas modeled with the E54K mutation. Statistical analysis shows that interaction energies for tropomyosin and the mobile domain of troponin I were significantly more negative for E54K tropomyosin compared with that of WT tropomyosin (Figure 7, right). The more negative values suggest an increased association energy or affinity of the troponin I mobile domain to tropomyosin/actin. This is precisely the molecular effect that reproduced the main features of steady-state and twitch activity in our simulations of E54K.

### Confirming downstream pathogenic mechanisms in mutant EHTs.

It is notable that even our best-performing simulations tended to underestimate peak twitch force changes in the case of E62Q (HCM) and overestimate them for E54K (DCM) (Figure 6). As we showed in previous work on *TPM1* variant S215L (19), we believe that this is because the model represents acute mutation effects only and does not account for their chronic impacts on cell size and morphology that emerge over time (Figure 4). Ultimately, peak force measured in EHTs is reflective not only of direct mutation changes in myofilament behavior but also due to downstream, chronic changes in myofilament content or arrangement within cardiomyocytes that exacerbate functional changes.

Thus, we hypothesize that counteracting the acute hyper- or hypocontractile mutational effects in EHTs should be sufficient to correct the chronic changes in peak force caused by cellular remodeling.

**Figure 8:**
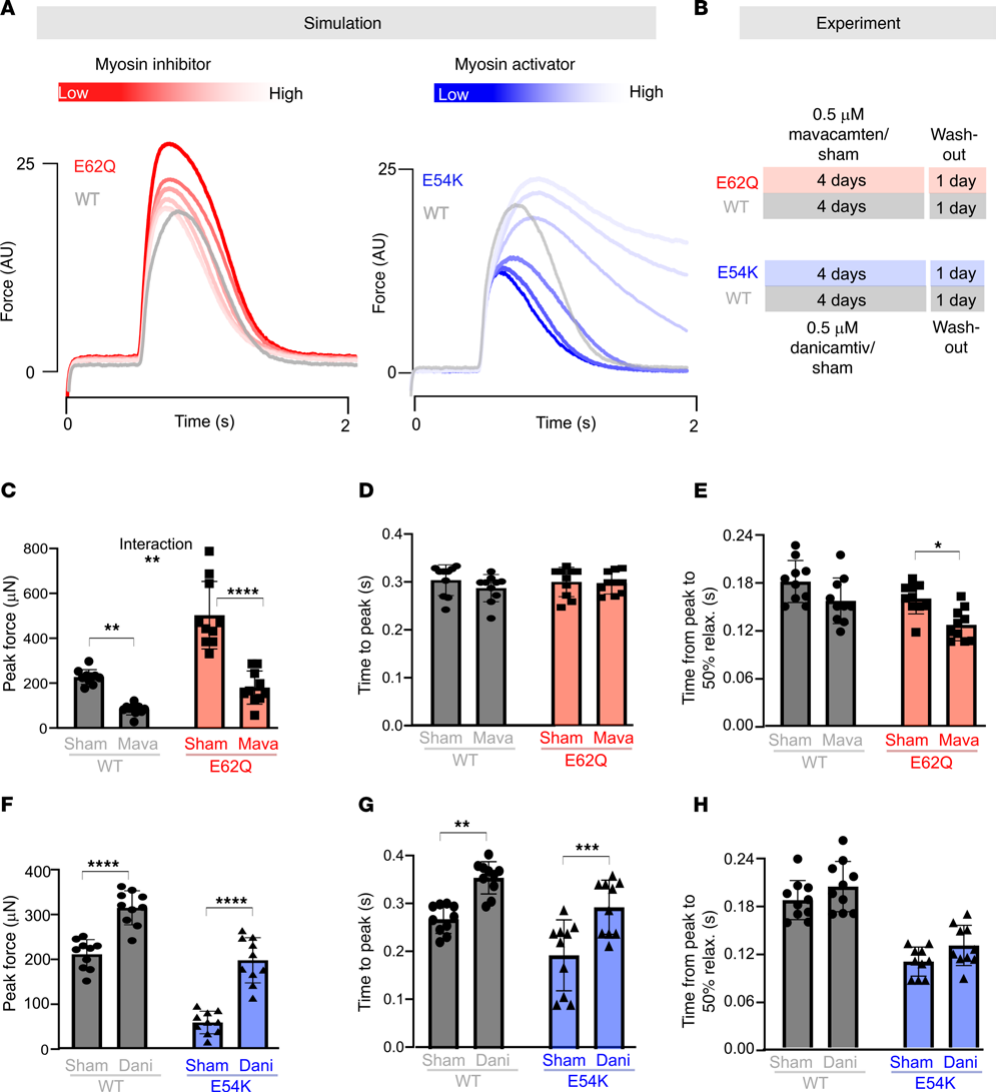
Drug treatment of EHTs. (**A**) Simulation of myosin modulators. (**B**) Experimental plan. (**C**–**E**) Peak force (**C**), time to peak (**D**), and time to relax to 50% (**E**) for E62Q and WT EHTs with mavacamten versus sham. (**F**–**H**) Peak force (**F**), time to peak (**G**), and time to relax to 50% (**H**) for E54K and WT EHTs with danicamtiv versus sham. Statistical analysis: 2-way ANOVA with multiple comparisons; *P* values for multiple comparisons are indicated using asterisks. *P < 0.05; **P < 0.01; ***P < 0.001; ****P < 0.0001.

To test this hypothesis, we turned to cardiac myosin–specific small-molecule modulators that provide a targeted means of either enhancing or inhibiting myofilament contraction. Because it was not certain that myosin modulators could rectify twitch force abnormalities caused by tropomyosin mutations, we first examined these scenarios in simulations of isometric twitches. We represented the action of myosin inhibitors or activators by adjusting the parameter for myosin binding to actin (f_XY_) (Figure 8A). The model predicted that a myosin inhibitor could effectively reverse the hypercontractile effects of the E62Q mutation, restoring the peak force to WT levels with minor reduction in time to peak. On the other hand, the hypocontractile twitch of a E54K mutation was reversed by a myosin activator, with some increase in relaxation time.

Confident that myosin modulators could appropriately counteract acute effects of the tropomyosin mutations, we proceeded to test whether non-acute application to mutant EHTs would reverse phenotypic abnormalities. We designed an experiment in which at the end of the usual culture period, we subjected our EHTs to 4 days of additional drug-supplemented media followed by a 24-hour washout (Figure 8B). We confirmed using liquid chromatography–mass spectrometry analysis that a 24-hour washout eliminated 85% of both compounds from drug-treated EHTs (Supplemental Figure 2). We observed that for E62Q, a 0.5 μM 4-day treatment with the myosin inhibitor mavacamten was able to reduce peak force to WT levels, with minimal changes in twitch kinetics. Similarly, a 0.5 μM 4-day treatment with the myosin activator danicamtiv was able to raise peak force to WT levels in E54K EHTs.

These results confirm our conclusion that EHT contractility reflects both chronic and acute mutation effects. If mutation-dependent changes in EHT peak force were solely due to the direct biophysical consequences of the amino acid substitution, the contraction phenotypes we originally observed should have been completely restored following washout of the myosin-modulating drugs. However, 24 hours after washout the peak forces produced in mutant tissues most closely resembled those of the non-mutant controls and had not returned to levels associated with the untreated mutants (Figure 8, C–H).

The other important conclusion furnished by this 4-day drug treatment is that it supports our proposed mechanistic pathways between each mutation and their resulting phenotype. That is, we assert that the E62Q mutation increases tropomyosin flexibility, destabilizes the blocked state of the thin filament, and consequently elevates contraction force beyond normal intrinsic levels, and finally provokes a cellular hypertrophic response. The link between hypercontractility and hypertrophy is confirmed by the fact that reducing contractility through mavacamten resulted in a durable reduction in twitch force in E62Q tissues. In like manner, the chronic hypocontractility measured in E54K mutant tissues was counteracted by administration of the myosin activator danicamtiv, which neutralized the tendency of E54K to suppress contraction.

## Discussion

In this study, we undertook a thorough multiscale analysis of two *TPM1* mutations associated with diverging ventricular phenotypes. The objective was to use this unique comparison to expose fundamental differences in the pathogenic mechanisms of HCM and DCM. Our data provide support to the paradigm that mutations that cause intrinsic elevation of actin-myosin contractility provoke cardiomyocyte hypertrophy, while those that intrinsically decrease contractility lead to cardiomyocyte lengthening without hypertrophy (4, 20, 21). Unbiased gene expression data confirm extensive differential pathway activation stemming from the distinct biophysical changes induced by E62Q and E54K within tropomyosin and the thin filament complex. Small-molecule treatments that neutralized these respective mutation-induced defects dramatically resolved chronic contractile phenotypes. To our knowledge, this is the first time HCM and DCM mechanisms have been directly compared at this level of detail and within a human context.

The HCM-causing mutation E62Q and the DCM-causing mutation E54K have been studied extensively in previous work. However, in many aspects these studies report conflicting interpretations or fail to establish a clear mechanism. E62Q was first reported in 2003 linked to several cases of HCM in a multigeneration Dutch family with an estimated penetrance of 75% and disease frequency of 0.2% (22). Subsequent studies have shown E62Q tropomyosin to confer greater sensitivity to calcium and an impaired ability to inhibit actomyosin ATPase activity (23, 24). While some studies have found evidence of weakened actin–E62Q tropomyosin interaction (10, 11, 25), others found no difference in F-actin binding affinity of E62Q using actin-tropomyosin co-sedimentation assay (26). In silico studies have previously shown higher global flexibility of E62Q tropomyosin, and decreased persistence length (25). In our EHTs, E62Q showed a 3-fold increase in peak force without any changes in kinetics and no difference in the length-dependent activation. The hypercontractile feature (19, 27–29) and impaired Frank-Starling mechanism (19, 28) are common to many other reported variants of HCM. Similar to previous studies with HCM-associated tropomyosin mutants E192K and S215L (19, 27), E62Q showed an increase in diastolic stiffness. Atomistic simulations of tropomyosin showed that each of these mutations caused bending stiffness of the molecule to be less than wild type. In the context of thin filament regulation, reduced tropomyosin stiffness prevents cooperative inhibition of myosin binding sites on actin under low Ca^2+^ conditions, leading to residual cross-bridge activity during the diastolic interval. At the level of the intact heart, it is therefore anticipated that reduced tropomyosin stiffness would lead to greater resistance to ventricular filling.

By contrast, E54K was identified as a novel mutation in 2001 in a study involving DCM patients (30). Several studies have shown a decrease in E54K tropomyosin calcium sensitivity (12, 23, 31), while others have reported no change compared with WT (6, 32). These biophysical changes have proven to have physiological consequences. Transgenic mice expressing E54K tropomyosin demonstrated impaired systolic and diastolic function (13). In our EHTs, E54K exhibited strikingly different isometric twitch profiles from E62Q. In the case of E54K, we observed a hypocontractile, short-lived twitch with essentially no length-dependent activation. This is reminiscent of the study by Schwinger et al. in which skinned muscle fibers obtained from terminally failing myocardium with DCM showed complete failure to exhibit the Frank-Starling mechanism (33). In contrast with HCM-linked variants, the DCM-associated E54K tissues were very compliant. This reduced passive stiffness may be due to a shift toward the longer, more compliant N2BA isoform of titin that is predominant in DCM (34, 35).

The drastic differences in functional phenotypes encouraged us to look at the transcriptomic changes that were present in mutant tissues. Bulk RNA sequencing and mass spectrometry analysis revealed distinct transcriptomic and proteomic signatures in HCM and DCM. Because transcriptional changes do not always result in protein-level changes, we focused on genes and proteins that were regulated in tandem. Independent analysis of both data sets revealed a handful of upstream transcriptional regulators that were synonymously altered. In HCM, both transcriptional and proteomic data sets suggested activation of *ARID1A*- and *TEAD2*-associated genes and inhibition of *RB1*-associated genes. *ARID1A* plays a role in ischemic heart disease by suppressing cardiac proliferation (36). Inhibition of *RB1* has been shown to induce cardiac hypertrophy (37). *TEAD2* (also known as transcriptional enhancer factor, or TEF-2) plays a key role in muscle differentiation and development. Several kinases involved in hypertrophic pathways such as protein kinase A and protein kinase C can posttranslationally modify TEAD proteins (38, 39). *TEAD2* therefore may play an important role in HCM and DCM disease progression. In DCM EHTs, *MYC*- and *NFE2L2*-associated genes were activated, while *SNAI2*- and *NANOG*-associated genes were inhibited. The proto-oncogene *MYC* encodes c-Myc, which is a potential therapeutic target for cardiomyopathies (40). *NFE2L2* is the gene encoding the transcription factor NRF, which mediates oxidative stress response and plays a key role in cardiac protection (41). *SNAI2* is a transcription factor that is crucial for atrioventricular development (42).

Several other pathways were shown to be altered by HCM/DCM in the analysis of proteomic and transcriptomic data, such as oxidative phosphorylation, fibrosis, and mitochondrial dysfunction, all of which have previously been differentially regulated in cardiomyocytes with altered aspect ratios (15). This encouraged us to look at potential changes in cell sizes in mutant cardiomyocytes. 2D stained images demonstrated that even in the absence of hormones, hemodynamic load, or adrenergic stimulus, E62Q cells exhibited a sharp increase in cell size while E54K cells had an elongated morphology. It has been stipulated before that pathological remodeling in adult hearts in response to chronic pressure overload results in concentric hypertrophy where sarcomeres are added in series while a chronic volume overload would result in eccentric hypertrophy where sarcomeres are added in parallel (43). However, it may be possible that cardiomyocytes have an intrinsic force-morphology homeostatic pathway that is independent of hemodynamic or adrenergic regulation.

Having characterized the divergent functional, transcriptomic, and morphological changes that occur as a result of E62Q and E54K mutations, we then focused on identifying the most immediate biophysical impact of the genetic insult. To that end, we devised several hypothetical changes in model molecular parameters through which mutant tropomyosin might impact thin filament regulation and tested them using our computational model. For the HCM mutant E62Q, the hypothesis that best matched our experimental data was a drop in tropomyosin stiffness combined with an increase in blocked-closed equilibrium constant. Consistent with these model parameters being key to the phenotype, atomistic simulations indicate that E62Q can reduce effective tropomyosin stiffness. Furthermore, in the blocked state E62 engages in interactions with actin residues, which stabilize the off state. These interactions would either be weaker or more transient or may not be formed altogether by Q62; thus, an E62Q-induced destabilization of the blocked state can lead to an increase in the blocked-closed equilibrium constant, as predicted by our comprehensive modeling.

In the case of E54K, unlike E62Q, there are no direct contacts between actin or troponin and tropomyosin in the structure; therefore, the pathogenic mechanism likely propagates via long-range allosteric interactions that result in an increase in the association rate of the troponin I mobile domain to tropomyosin/actin. To our knowledge, this proposed mechanism for E54K has never been suggested before for any DCM tropomyosin mutant. Local residue changes to the coiled-coil structure of tropomyosin can result in long-range alterations in tropomyosin structure even up to a few hundred angstroms away from the original site of the mutation (11, 44, 45). Molecular dynamics simulations show that E54K tropomyosin–troponin I interactions are more stable in the B state compared with WT tropomyosin–troponin I interactions, validating this notion (Figure 7, right).

Interestingly, however, given the likelihood of a generic force sensing mechanism inherent to cardiomyocytes, the specific mechanism driving the pathogenicity may not be consequential in terms of treatment. We tested this theory in our computational models by altering myosin activation/inhibition to correct the phenotype and observed a reversal in the contractility. A 4-day treatment with myosin activators/inhibitors was able to achieve phenotype rescue even after 48 hours of washout, which explains the success of small-molecule myosin modulators in a clinical setting regardless of underlying causes of HCM or DCM (46–49).

Our study is certainly not without limitations. Although EHTs made from human iPSC–derived cardiomyocytes offer an excellent testing bed for many physiologically relevant questions, the isolated system lacks hemodynamic load, or adrenergic control, which can impact tissue function. Our proposed hypotheses only represent a subset of myriad possible mechanisms that we either assumed to be unlikely or are not easily implementable using our existing model. The best fit for our computational simulations was matched to literature data from in vitro experiments that have their own limitations, and values may not be comparable across studies. Previously, we showed that there was a linear correlation between gene dosage and phenotypic severity of TPM1 mutation S215L (19). It is important to note that in this study we tested only homozygous mutations in order to accentuate the differences. We anticipate that the more clinically relevant heterozygous mutation would behave similarly but have a milder effect. Moreover, this study only analyzes one HCM and one DCM mutation in depth. Future work should involve testing this paradigm on a broader range of thin filament mutations.

Despite the limitations, this study clearly demonstrates divergent trends in E62Q and E54K mutants across several spatial scales and uncovers important insights. The first is that mutant cardiomyocytes alter their morphology likely as a result of an inherent force sensing mechanism that detects a departure from a homeostatic set point. Secondly, there are key upstream regulators that may be responsible for driving the transcriptomic changes at an early stage. Thirdly, the two mechanisms likely propagate via very different mechanisms. And finally, a mechanism-agnostic approach is effective in rescuing the phenotype.

## Methods

Further information can be found in Supplemental Methods.

### Sex as a biological variable.

Sex was not considered as a biological variable in this work. All experiments were conducted using 3 isogenic cell lines originally derived from a human male patient (GM23338, Coriell Institute).

### iPSC maintenance and differentiation.

Isogenic cell lines were created by the University of Connecticut Health Sciences Human Genome Editing Core, using CRISPR/Cas9 to introduce the *TPM1^E62Q/E62Q^* and *TPM1^E54K/E54K^* variants into a commercially available WT human induced pluripotent stem cell (iPSC) line (GM23338, Coriell Institute). Upon delivery, the iPSCs were differentiated into ventricular cardiomyocytes (50). First, iPSCs were plated on a well coated with Matrigel (Corning; 1:60 diluted) for culture for 3–4 days in mTeSR medium (Stemcell Technologies, 05850) until approximately 90% confluence and treated with 20 μM CHIR99021 (Selleckchem) on day 0 for 24 hours and 5 μM IWP4 (Stemgent) on day 3 in RPMI medium (Gibco)/B27 (Gibco) minus insulin medium for 48 hours. During the cardiac differentiation, media were changed every other day with RPMI medium/B27 minus insulin medium. After beating, cardiomyocytes were cultured in regular RPMI medium/B27 (including insulin). Cardiomyocytes at day 14 were treated with 4 mM lactate (MilliporeSigma) in glucose-free medium for 4 days to obtain enriched cardiomyocytes.

### Cell size measurements.

Differentiated cardiomyocytes at day 18 were passaged at a low concentration onto Matrigel-coated 12-well tissue culture plates. The cells were cultured in RPMI medium/B27 (including insulin) for 4 days, after which they were fixed using 4% paraformaldehyde overnight at 4°C before immunostaining. The primary cardiac troponin T antibody used was MS-295-p0 (Thermo Fisher Scientific) at a dilution of 1:500. The secondary antibody used was goat anti-mouse IgG–Alexa Fluor 488 (Thermo Fisher Scientific, A-11029) at a dilution of 1:300. The cells were incubated for 1 hour in secondary antibody and Hoechst dye (NucBlue, Thermo Fisher Scientific) before imaging under an inverted fluorescent microscope at ×40. Images were analyzed using ImageJ (NIH) to calculate area and aspect ratio for each cell. ImageJ calculates aspect ratio as the ratio of major axis to minor axis of the fitted ellipse.

### Fabrication of engineered heart tissues.

Engineered heart tissues (EHTs) were made by seeding of iPSC-cardiomyocytes (iPSC-CMs) into decellularized porcine myocardial slices according to our previously published protocol (51). Briefly, 150-μm-thick slices were obtained from porcine left ventricular free-wall blocks, mounted onto custom tissue culture cassettes, and decellularized. The scaffolds were then treated with 10% FBS and 2% antibiotic-antimycotic (Thermo Fisher Scientific) overnight. iPSC-CMs were dissociated using TrypLE Express 1X (Thermo Fisher Scientific) followed by washing with PBS and manual pipetting. For this study, we seeded WT, E62Q, and E54K cell suspensions containing 1 million cells onto each scaffold and allowed them to incubate overnight. The EHTs were subsequently grown in DMEM plus 2% B27 plus insulin for 14 days. EHTs were then harvested for RNA sequencing or evaluated mechanically.

### RNA sequencing.

For RNA extraction, tissues were flash-frozen and crushed with plastic pestles. The aqueous phase was collected after TRIzol (Ambion) phase separation, and the RNA pellets were precipitated for total RNA extraction. Samples were treated with DNase and cleaned with QIAGEN RNeasy columns. Samples were sequenced by Yale Center for Genomic Analysis (Illumina HiSeq 2500; multiplexed, paired-end reads of 100–base pair length with 25 million reads per sample). Data analysis was performed on Partek Flow. Alignment was performed using STAR 2.7.8a (https: //github.com/alexdobin/STAR/releases) and hg38 as the reference genome. Samples were clustered by average linkage using Pearson’s dissimilarity. Differential analysis was performed using DESeq2 (Bioconductor), and genes with *P* less than 0.05 and fold change less than or greater than 1.5 were filtered. Pathway analysis was performed using Ingenuity Pathway Analysis software (QIAGEN) with the filtered data set. Top relevant canonical pathways with a *P* less than 0.05 were considered. Upstream analysis was also performed using QIAGEN Ingenuity Pathway Analysis software, and top transcription factors with the highest *z* score and lowest *P* values were identified.

### Mass spectrometry.

EHTs were resuspended in 9 M urea (MilliporeSigma) and sonicated. Protein concentration was determined using a bicinchoninic acid assay (Thermo Fisher Scientific). One hundred micrograms of protein per sample was used for in-solution digestion. Samples were reduced with 5 mM DTT for 45 minutes and then alkylated by incubation in 10 mM iodoacetamide for 30 minutes. The proteins were digested next with 5 μg trypsin/LysC protease for 18 hours at 37°C. The reaction was stopped by addition of trifluoroacetic acid (TFA) at 0.1% final volume to bring the pH under 3. Samples were then dried by vacuum centrifugation, reconstituted in buffer A (3% acetonitrile, 0.1% formic acid), and analyzed by liquid chromatography–tandem mass spectrometry.

Purified peptide concentration was measured using Pierce Quantitative Peptide Assay (Thermo Fisher Scientific), and 1.0 μg was loaded onto a Vanquish Neo UHPLC system (Thermo Fisher Scientific) with a heated trap and elute workflow with a C18 PrepMap, 5 mm. Spectra were acquired with an Orbitrap Eclipse Tribrid mass spectrometer with FAIMS Pro interface (Thermo Fisher Scientific). Raw data were analyzed with Proteome Discoverer 2.5 (Thermo Fisher Scientific) using SEQUEST-HT search engines. The data were searched against the *Homo sapiens* UniProt protein sequence database (UP000005640). The search parameters included precursor mass tolerance of 10 ppm and 0.6 Da for fragments, allowing 2 missed trypsin cleavages. Oxidation (Met), acetylation (protein N-term), and phosphorylation (+79.966, STY) were set as variable modifications, and carbamidomethylation (Cys) as a static modification. Normalization was based on total peptide amount; low-abundance peptides were removed by filtering out of proteins with less than 3 peptide spectrum matches. Protein abundance calculations were based on the summed abundance of the connected peptide groups, and protein ratio was calculated as the median of all possible peptide ratios between replicates of all connected peptides. A 2-tailed *t* test was used for class comparison.

### Active and passive mechanical measurements.

EHTs were assessed for their active contraction mechanics using a custom-built setup that utilized a World Precision Instruments (WPI KG7) force transducer. The EHTs were kept in Tyrode’s solution at 36°C and 7.4 physiological pH as they were paced at 1 Hz and stretched from culture length (0% stretch) to up to 10%. The resulting isometric twitch was analyzed in MATLAB (MathWorks) to calculate peak force, time from stimulus to peak contraction, time from peak to 50% relaxation, and normalized tension-time integral. To characterize intracellular calcium dynamics, some EHTs were loaded with the ratiometric fluorescent indicator Fura-2 AM (Millipore) by incubation at room temperature for 20 minutes in loading solution (Tyrode’s solution with 17 μg/mL Fura-2 AM, 0.2% Pluronic F127, and 0.5% Cremophor EL) and subsequently imaged at 36°C using a photometric system as previously described (51).

For passive mechanical measurements, EHTs were preconditioned by stretching them repeatedly, from slack length (–3% stretch) to a maximum stretch of 9% for 3 cycles at a rate of 0.015 mm/s (0.25% muscle length/s). After preconditioning, EHTs were paced at 1 Hz, and the force was recorded as they were stretched from –3% to 9%. The diastolic force produced was extracted by a custom MATLAB script as previously described (52). To assess the effect of acute mavacamten treatment on EHTs, passive diastolic force was measured before addition of mavacamten. Tissues were incubated at 36°C in 2 μM mavacamten in Tyrode’s solution for 30 minutes while being paced at 1 Hz. Passive diastolic force was measured again after incubation once the active force had equilibrated.

Optical coherence tomography scans were performed on tissues to capture the cross-sectional area, which was then calculated using ImageJ. The cross-sectional area was used to normalize the passive force measured and therefore calculate the passive stress of the tissues.

### Computational simulations.

We used a 24-state Markov model previously described by Creso and Campbell to predict changes in steady-state and isometric twitch conditions (16). Briefly, the model simulates the behavior of 26 regulatory units in series, tracking for each unit the states of tropomyosin and key domains of troponin C and troponin I. The simulations use either a steady-state Ca^2+^ concentration or a transient to elicit contractile activity over a set time interval. Force was output as the number of regulatory units in a myosin-bound state.

To simulate in vitro motility assay data, steady-state force values at various Ca^2+^ concentrations were obtained by simulation of a 5-second interval to ensure attainment of steady-state conditions. The steady-state force was determined by averaging of force over a window of the final 25% of each simulation. To convert these force values into sliding filament velocity, a simple proportionality (effective filament viscosity) between force and velocity was assumed (16). Steady-state force values at different Ca^2+^ concentrations were used to produce velocity-pCa plots, which were fit using the Hill equation. For WT steady-state simulations, model parameters were chosen such that the steady-state behavior matched previous literature in terms of pCa_50_ and n_H_ (11, 12). Next, for each hypothesis being investigated, only the relevant parameters were changed until the mutant steady-state behavior in the literature was replicated. For a few of the hypotheses that involved a single parameter change, there was no solution that perfectly matched both pCa_50_ and n_H_ literature values, and in those cases the parameter change reflecting the closest match to pCa_50_ was selected. For each hypothesis, we recorded the percentage change in the parameters that yielded best match to literature in terms of steady-state behavior.

For isometric twitch simulations of WT, parameter sets were chosen such that twitches recapitulated characteristics similar to those seen with WT EHTs previously described in the literature (16, 19). Then we applied the same proportional changes in parameters recorded earlier for each competing hypothesis to predict isometric twitch forces. The complete parameter sets are shown in Table 2. Twitch events were simulated by allowing the system to reach steady state at a diastolic Ca^2+^ concentration of 0.1 μM. The Ca^2+^ concentration was then allowed to produce a transient by increasing up to 1 μM based on data from Stull et al. (53).

Model scripting and data post-processing were conducted in MATLAB, while the Markov chain–Monte Carlo algorithm was implemented in CUDA C++ for parallel processing. Simulations were executed on an Nvidia GeForce RTX 2080Ti graphics processing card.

To ensure convergence of the stochastic model, the simulation time course was repeated 1,920 times on the graphics processing unit (GPU), and the average force at each time step was calculated. To further reduce stochastic noise, twitch simulations were run 10 times and averaged to calculate twitch properties (diastolic force, peak force, time to peak force, and time to 50% relaxation).

### Molecular dynamics.

Molecular dynamics simulations of isolated tropomyosin dimers were performed as previously described (54). The starting model was an ideal coiled-coil backbone where the tropomyosin side chains had been built to match crystal structure coordinates (55, 56). After the E62Q mutation was incorporated, the structure was minimized in CHARMM (https://academiccharmm.org/) using the CHARMM 27 force field and the GBSW implicit solvation model. Simulations were then run for 30 nanoseconds at 300 K with a frictional drag of 1 per picosecond applied to solvent-exposed heavy atoms, a time step of 2 femtoseconds, and the SHAKE algorithm. The local superhelical changes in tropomyosin flexibility (δ angle) were calculated in CHARMM as previously described (54).

For simulations of E54K on an actin filament, the mutation was incorporated into the starting WT model that had been subjected to 30 nanoseconds of simulation as previously described (19) consisting of 28 actin monomers, 8 tropomyosin chains, 4 troponin T chains, and 2 troponin I chains in explicit solvent containing 0.15 M NaCl and 3 mM magnesium chloride with the Mutator plug-in in VMD (57). The solvated E54K system was then minimized in NAMD (58) in the CHARMM 36 force field. For simulation, the system was then heated to 300 K over 30 picoseconds using Langevin temperature control and equilibration over the next 500 picoseconds at constant volume with gradual release of constraints on protein atoms. At this point, a short run of 100 picoseconds was performed with the Langevin piston turned on to bring the system pressure up to 1 atm. For the production runs, the barostat was turned off, a constraint on the non-surface actins was applied throughout with a force constant of 0.1 kcal/mol/Å^2^ to maintain actin filament structure, and the simulation was run at constant volume for 30 nanoseconds and the results compared with the WT simulation as run from 30–70 nanoseconds. Interaction energies between tropomyosin and actin were calculated using the NAMDEnergy plug-in in VMD with default settings. E54K simulations were performed on 3 separate B-state runs, giving a total of 6 independent tropomyosin copies for analysis.

### Drug treatment.

For treatment of EHTs with drug, EHTs were grown to 21 days in DMEM and then subjected to 4 days of media supplemented with 0.5 μM mavacamten, 0.5 μM danicamtiv, or DMSO vehicle, changed every 2 days. On the fourth day, medium was removed, and EHTs were washed twice with drug-free medium. EHTs were then grown in these drug-free medium for 24 hours before functional testing was conducted as before.

### Statistics.

Results are given as the mean with standard deviation. For a comparison of 2 groups, statistical significance was determined using 2-tailed Student’s *t* test with a confidence level of *P* less than 0.05. For paired analysis with repeated measurements on the same samples, paired *t* tests were used with a confidence level of *P* less than 0.05. For comparison of groups under a single condition, 1-way ANOVA was used followed by pairwise comparison using post hoc testing with Tukey’s correction to determine significant differences with a confidence level of *P* less than 0.05.

For comparison of groups under multiple conditions, 2-way ANOVA was used followed by pairwise comparison using post hoc testing with Tukey’s correction to determine significant differences with a confidence level of *P* less than 0.05.

Bootstrap analysis (59) was performed to analyze the molecular dynamics data in Figure 7 (right). This analysis was run on MATLAB (code adapted from https://courses.washington.edu/matlab1/Bootstrap_examples.html) with 10,000 repeated samplings.

### Study approval.

This study involved no animal testing and therefore did not require any approvals.

### Data availability.

All data generated or analyzed during this study are included in this published article and its supplementary information files. RNA sequencing data can be found on the Gene Expression Omnibus (GEO) database (https://www.ncbi.nlm.nih.gov/geo/) under accession ID GSE251993. Mass spectrometry data can be found on the MassIVE repository (https://massive.ucsd.edu/) under data set identifier MSV000095377. Raw data for each experiment can be found in the Supporting Data Values file.

## Author contributions

SSH, LRS, SGC, JRM, WL and JAK contributed to the study design. SSH, MJR, LK, MEB, and AGAZ contributed to data collection and analysis. All authors participated in writing and revising the manuscript.

## Supplementary Material

Supplemental data

Supporting data values

## Figures and Tables

**Table 1:**
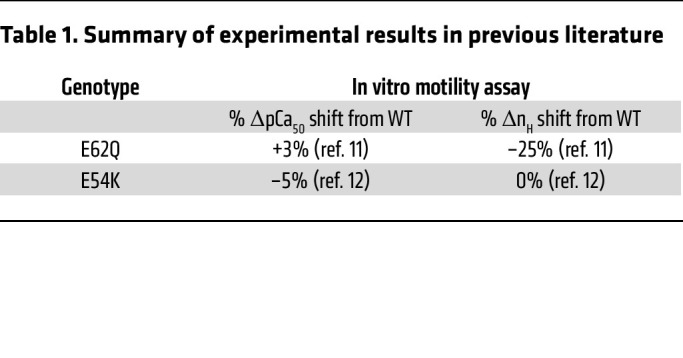
Summary of experimental results in previous literature

**Table 2:**
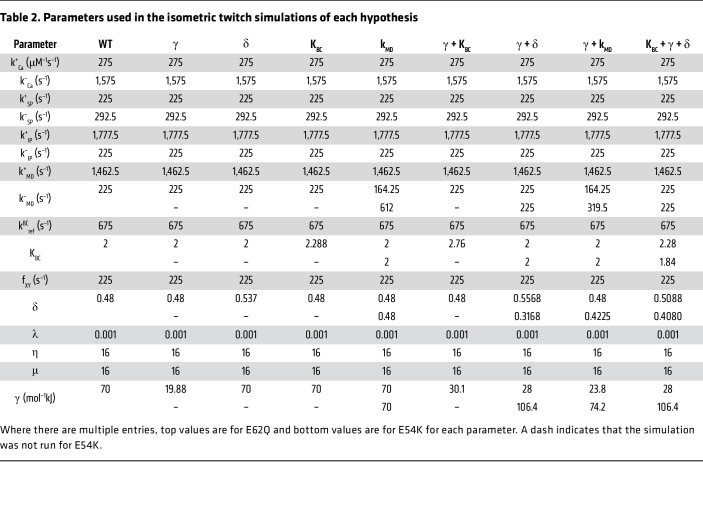
Parameters used in the isometric twitch simulations of each hypothesis

## Version 1 10/22/2024

In-Press Preview

## Version 2 12/16/2024

Electronic publication
