# Supplementary material for: Distinct mechanisms drive divergent phenotypes in hypertrophic and dilated cardiomyopathy–associated TPM1 variants
Source: J Clin Invest. 2024 Dec 16;134(24):e179135. doi: 10.1172/JCI179135 (PMC11645150; doi:10.1172/JCI179135)
Supplement: Supplemental data [file jci-134-179135-s038.pdf]

The Journal of Clinical Investigation

Supplementary Information for

Distinct Mechanisms Drive Divergent Phenotypes in Hypertrophic and Dilated Cardiomyopathy associated *TPM1* variants

Saiti S Halder[1], Michael J Rynkiewicz[3], Lynne Kim[1], Meaghan E Barry[2], Ahmed GA Zied[4], Lorenzo R Sewanan[1], Jonathan A Kirk[4], Jeffrey R Moore[2], William Lehman[3], Stuart G Campbell[1]

1. Department of Biomedical Engineering, Yale University
2. Department of Biological Sciences, University of Massachusetts Lowell
3. Department of Pharmacology, Physiology, & Biophysics, Boston University Chobanian & Avedisian School of Medicine
4. Department of Cell and Molecular Physiology, Stritch School of Medicine, Loyola University Chicago

Corresponding Author:

Stuart G Campbell
stuart.campbell@yale.edu
10 Hillhouse Avenue

PO Box- 208267
New Haven, CT- 06520-8267

This PDF file includes:

Supplementary text
Figures S1 to S3

Results

E62Q and E54K calcium characteristics are consistent with previous literature

Farman et al 2018 and Bai et al 2012 previously reported E62Q and E54K calcium characteristics. Because these experiments were done using different experimental setup, we wanted to confirm that these mutants would exhibit the same characteristics as was previously reported. We therefore performed a regulated in-vitro motility assay to compare the calcium characteristics of these two mutants (Figure S1). In our system the E62Q mutant exhibited a 3% increase in the pCa_{50} and E54K showed a 7.6% decrease in the pCa_{50}. These results are consistent with the behavior observed in Farman et al, 2018 and Bai et al, 2012.

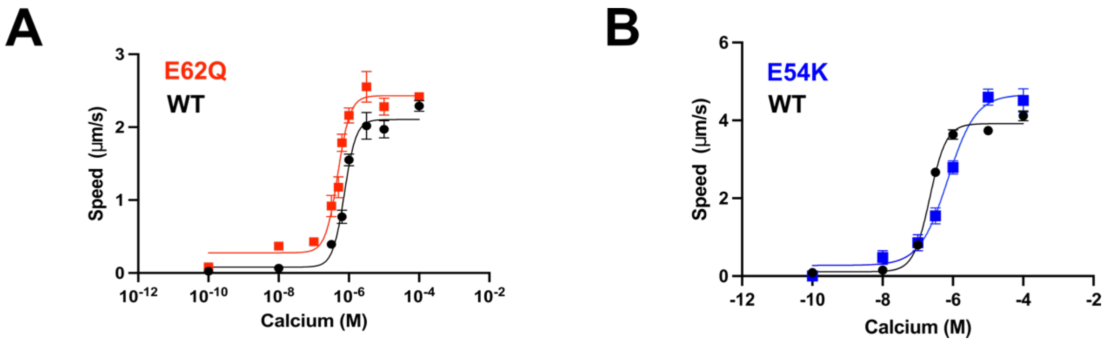

Fig. S1. Regulated In-Vitro Motility Assay. (A) In vitro motility assay showing increase in Ca sensitivity for E62Q ($p < 0.001$) and (B) In vitro motility assay showing increase in Ca sensitivity for E54K (p-value = 0.0259)

Majority of both drugs are washed out in 24 hours

We wanted to know if the 24-hour washout following a 4-day drug treatment is adequate. To that end, we performed an experiment where we subjected wild type EHTs to 4 days of either mavacamten or danicamtiv treatment. We then measured the amount of drug in our EHTs prior to washout and at varying time points after washout. The results of this study showed that on average 85% of the drug was washed out in both cases following a 24-hour washout.

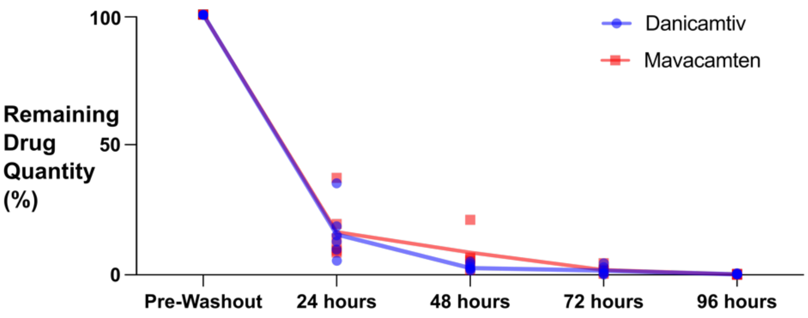

Fig. S2. Remaining Drug Quantity in EHTs. The amount of each type of drug (danicamtiv and mavacamten) remaining in the tissues after washout. For this experiment n=6 for each drug and each time point tested.

Query of sarcomeric proteins from mass spectrometry data

From the mass spectrometry data, we queried sarcomeric proteins to know the sarcomeric protein content of our EHTs. Our EHTs exhibited no significant changes between mutant EHTs and WT EHTs in terms of *MYH6*, *MYH7*, *TNNI1, TNNI3 , TTN* and ratio of cardiac troponin I to total troponin I and beta myosin heavy chain to total myosin. HCM EHTs had significantly higher *MYL2* compared with WT EHTs (FigureS3).

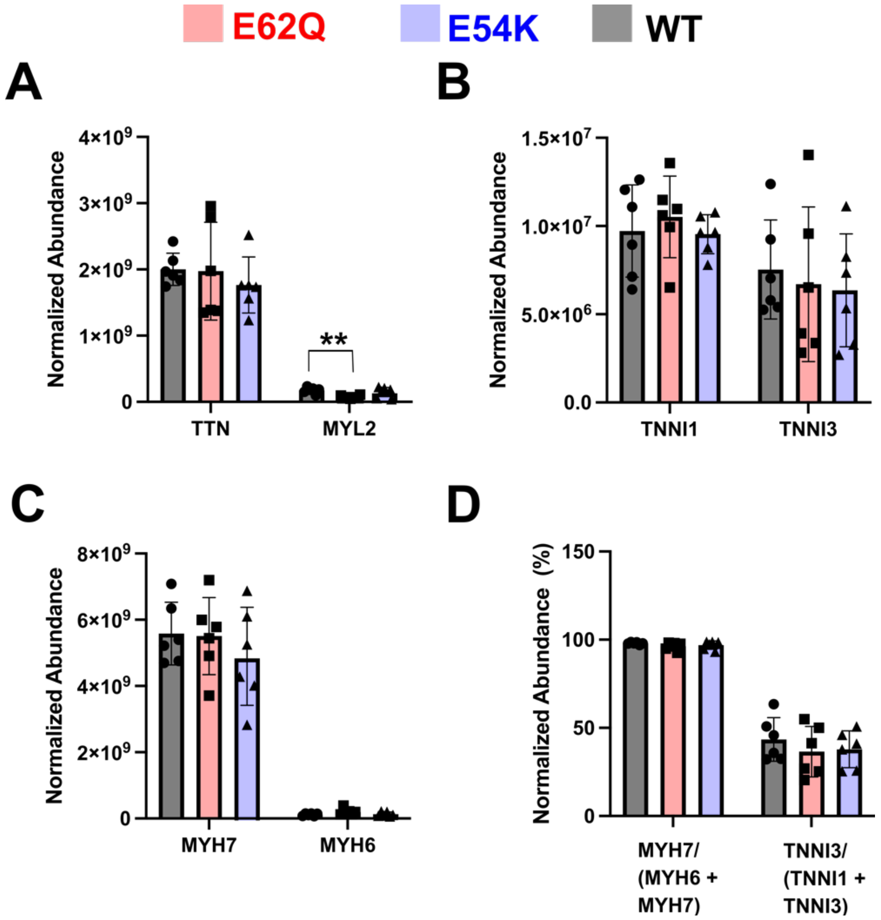

Fig. S3. Sarcomeric Protein Quantity in EHTs. A) Relative protein abundances of TTN and MYL2 B) Relative protein abundances of TNNI1 and TNNI3 C) Relative protein abundances of MYH7 and MYH6 D) Relative Abundance of MYH7 and TNNI3 as a % of total myosin and troponin I respectively

Methods

Regulated In-Vitro Motility Assay

After expression, tropomyosin was purified from cell extracts via three rounds of isoelectric precipitation and two rounds of ammonium sulfate cuts at 55% and 70%, respectively. Purified protein was ultimately dialyzed into storage buffer (55mM KCl, 25mM BES, 4mM $MgCl_2$, 5mM EGTA, 1mM DTT, pH 7.4) and stored at -20°C. Protein purity was confirmed via SDS PAGE.

In vitro motility assay was conducted in a nitrocellulose-coated flow chamber as described previously (Sundar et al., 2020). Briefly, chicken skeletal muscle myosin, diluted to 50ug/ml in myosin buffer (300 mM KCl, 25 mM BES, 5 mM EGTA, 4 mM $MgCl_2$, pH 7.4, 0.1 M DTT) was added to a nitrocellulose coated flow chamber and incubated for 1 min. Once incubated, the unbound surface was blocked with 1 mg/mL BSA in myosin buffer by adding 25µl to the flow cell and incubating for 1 minute. Unbound protein was removed by washing the chamber with 25µl of actin buffer (55 mM KCl, 25 mM BES, 50 EGTA, 4 mM $MgCl_2$, pH 7.4, 0.1 M DTT). Unlabelled actin was added to the flow cell and incubated for 2 minutes to allow the actin to bind to the surface-bound catalytically inactive myosin heads, unbound actin was removed with an actin buffer wash of 25 µl actin buffer with 1mM ATP followed by ATP removal with four washes in actin buffer without added ATP. TRITC-phalloidin labelled F-actin with 800 nM tropomyosin and troponin was added to the flow cell and incubated for 5 minutes. Movement was assessed after the addition of different concentrations of Ca^{2+} with motility buffer (40 mM KCl, 25 mM BES, 5 mM EGTA, 4 mM MgCl2, pH 7.4) in the presence of 1 mM ATP and oxygen scavengers (45 mg/mL catalase, 10 mg/mL glucose oxidase, and 40 mg/mL dextrose/glucose). Four to six videos at each pCa of the motile thin filaments were recorded and later analysed using the MTrckJ ImageJ plugin. All experiments were repeated as triplicates and the values were averaged to determine filament velocity and fraction of filaments motile.

Measuring Drug Concentration After Washout

We designed a study where we subjected our EHTs to the same initial drug concentration of 0.5 µM and variable washout times. Individual EHTs were homogenized using a bead mill homogenizer,(Bead Rupture Elite-Omni International). Tissue samples were pulverized in a solution of 70:30 2-propanol:water containing five to eight 2.4mm stainless steel beads with agitation, to extract the test articles and produce a homogenate amenable to analysis via Liquid Chromatography. 150uL of 70:30 2-propanol:water was added to each tissue sample, after individual weights were measured, and homogenized. A 25 µL sample aliquot of sample was transferred to a 96-well plate and combined with 50µL of HPLC grade acetonitrile containing internal standard (5ng/mL tolbutamide), or pure acetonitrile or matrix blanks. The samples were vortex mixed at 850 rpm for 5 minutes, then centrifuged at 3000 rpm for 5 minutes. Following centrifugation, 30µL of supernatant were transferred to a clean 96-well plate and combined with 70µL of HPLC grade water. Samples were vortex mixed and transferred to an LCMS for analysis.
